# Association of Glomerular Complement C4c Deposition With the Progression of Diabetic Kidney Disease in Patients With Type 2 Diabetes

**DOI:** 10.3389/fimmu.2020.02073

**Published:** 2020-09-02

**Authors:** Suyan Duan, Lianqin Sun, Guangyan Nie, Jiajia Chen, Chengning Zhang, Huanhuan Zhu, Zhimin Huang, Jun Qian, Xiufen Zhao, Changying Xing, Bo Zhang, Yanggang Yuan

**Affiliations:** Department of Nephrology, The First Affiliated Hospital of Nanjing Medical University, Nanjing Medical University, Nanjing, China

**Keywords:** complement, C4, progression, diabetic kidney disease, renal pathology

## Abstract

**Objectives:** As accumulating data supporting the potential role of the complement system in the pathogenesis of diabetic kidney disease (DKD), the present study aimed to explore the association of glomerular complement C4c deposition with the baseline clinicopathological characteristics and the prognosis of DKD in type 2 diabetes (T2DM) patients.

**Methods:** A total of 79 T2DM patients with biopsy-proven DKD were enrolled. Clinicopathological features and renal outcomes were compared between groups divided by the glomerular C4c deposition patterns and median values of serum C4. Renal outcomes were defined by doubling of serum creatinine level or progression to end-stage renal disease (ESRD). A Cox proportional hazards model was employed to identify the risk factors associated with renal events.

**Results:** Patients with glomerular C4c deposition had worse renal insufficiency than those without C4c deposits, along with higher 24-h urinary protein, triglyceride, but lower serum albumin and higher interstitial inflammation score. Besides, serum C4 levels positively correlated with urinary protein and serum C3 levels. During 21.85 ± 16.32 months of follow-up, Kaplan-Meier curve analysis showed significantly faster deterioration of renal function for patients with positive glomerular C4c deposition as well as higher levels of serum C4. More specifically, more than 50% of the patients with glomerular C4c had co-deposition of C3c or C1q, and patients with glomerular complement complex of C4c and one or two of C3/C1q deposition had more severe proteinuria and a higher rate of DKD progression than those with negative C4c deposits. The univariate Cox regression indicated that factors of combined serum and glomerular C4, urinary protein, serum creatinine, serum C3, combined glomerular C4c and IgM and interstitial inflammation were associated with an increased risk of DKD, but only glomerular C4c intensity (HR 1.584, 95% CI [1.001, 2.508], *p* = 0.0497), as well as baseline age and diabetic neuropathy, were independent risk factors for renal survival by the multivariate Cox analysis.

**Conclusions:** Glomerular C4c deposition was associated with deteriorated renal function and outcomes in patients with T2DKD. Glomerular C4c deposition was an independent risk factor for DKD progression.

## Introduction

Diabetic kidney disease (DKD) is the most prevalent chronic kidney disease and is the major cause of the end-stage renal disease (ESRD) worldwide (1). In mainland China, an estimated 109.6 million adults have diabetes mellitus (DM) (2), and chronic kidney disease (CKD) associates with diabetes has become more prevalent compared to glomerulonephritis recently (3). Despite the availability of multiple drugs, the number of patients with diabetes who have ESRD continues to increase and that their prognosis is notably worse compared with those without diabetes (4). In recent years, immune-mediated inflammation, which is present throughout the development and is associated with the progression of DKD, has been considered to play a vital role in the pathogenesis of DKD (5, 6). Therefore, therapeutic strategies targeting inflammatory pathways have an attempt to slow the progression of DKD (7). As a part of innate immune system, the role of the complement system in DKD has come into focus, which promotes inflammation and heightens the clearance of impaired cells and pathogenic microbes from the body by phagocytic cells and antibodies (8).

Three different typical pathways can activate the complement system, including the classic pathway (CP), the lectin pathway (LP), and the alternative pathway (AP). As a 200 kDa glycoprotein, complement C4 is a principal element of the CP (9). Main attentions were focused on the LP in previous studies of the complement system in the DKD development. Two different mechanisms may contribute to the LP activation in the pathogenesis of DKD: sugars initiates LP activation and hyperglycemia-induced complement regulator dysfunction (9). C3 is activated by the C3-convertase C4bC2a when the LP is initiated. The C3-convertase formation relies on the former cleavage of C4 and C2. Therefore, one of the promising approaches to suppress the LP could block the C3-convertase by targeting C4 (10).

Several clinical studies have reported the association between complement component C4 and diabetes. Plasma C4 levels were associated with microvascular disease in diabetic patients (11). In addition, the serum C4 was significantly associated with the progression of Type 2 DKD (12). The complement components and relative fragment deposition are closely related to the pathogenesis of autoantibody-associated glomerulopathy, resulting in recruitment of infiltrating inflammatory pathways as well as direct glomerular injury (13). The previous study has demonstrated that the complement activation was involved in DKD and both glomerular deposition of C5b-9 and C4d were associated with the class of DKD (14). However, the role of glomerular C4 deposition in the progression of DKD is yet unknown.

The production of 2 fragments (C4a and C4b) which generates from the first cleavage of C4 have the ability to trigger cellular reaction (15). Then the second cleavage of the larger C4b fragment regulated by factor I with the company of C4bp (C4b-binding protein) results in 2 components, C4d and C4c (16). In the clinical practice of our center, glomerular C4c staining is routinely performed. Thus, our study aimed to investigate the association of glomerular deposition of complement C4c with clinical and pathological variables and renal progression in biopsy-proven T2DKD patients.

## Patients and Methods

### Subjects

**Figure 1:**
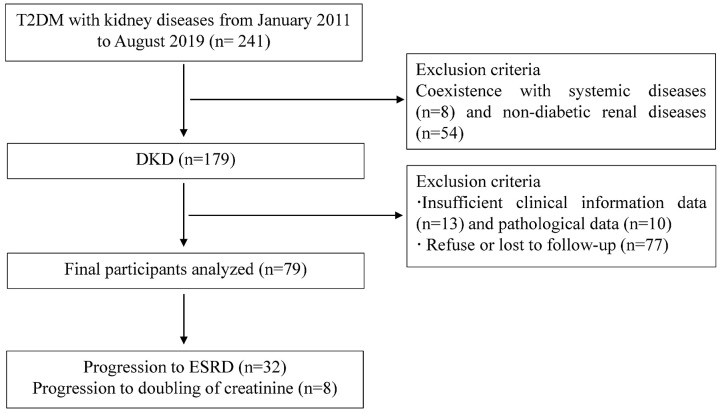
Flowchart of study participants. DKD, diabetic kidney disease; NDKD, non-diabetic kidney disease; ESRD, end-stage renal disease; T2DM, type 2 diabetes mellitus; LM, light microscopy.

A total of 241 T2DM patients with kidney diseases, diagnosed from January 2011 to August 2019 at the renal department of the First Affiliated Hospital of Nanjing Medical University, were retrospectively reviewed in this study (Figure 1). The inclusion criteria were patients with previously or newly diagnosed type 2 DM according to criteria established by the American Diabetes Association (ADA) in 2017 (17), and with chronic kidney disease (CKD) who had undergone renal biopsy pathological examination. The diagnostic criteria for CKD was defined as abnormalities of kidney structure or function, present for ≥3 months by KDIGO Clinical Practice Guidelines. The indications for renal biopsy were T2DM patients with renal damage who lacked absolute contraindications (i.e., inability to correct an obvious bleeding tendency, inability to correct severe hypertension, isolated kidney, active kidney infection, etc.), particularity T2DM patients without diabetic retinopathy (DR) or with obvious glomerular hematuria and/or short duration of T2DM, or with sudden onset of overt proteinuria, or sudden onset of low eGFR or rapidly decreasing eGFR, or >30% eGFR decline within 2–3 months of initiation of a renin-angiotension system inhibitor (18, 19). The diagnosis of DKD followed the Renal Pathology Society in 2010 (20). DKD was characterized by pathological features, consisting of thickened capillary basement membranes, glomerular hypertrophy and nodular mesangial sclerosis. The exclusion criteria were as follows: (1) coexistence of systemic diseases (autoimmune disease i.e., lupus nephritis, Henoch-Schönlein purpura nephritis, vasculitis, anti-glomerular basement membrane disease etc., malignancy); (2) biopsy-proven non-diabetic kidney diseases such as IgA nephropathy, membranous nephropathy, focal segmental glomerular sclerosis, etc.; (3) Type 1DM; (4) insufficient clinical information data (no information of complement values, 24-h urinary protein, etc.) or pathological data (<10 glomeruli for LM, or obtaining <10 glomeruli by renal biopsy, or no glomeruli or glomerular sclerosis in the immunofluorescence staining specimens etc.); (5) coexistence with acute renal injury or severe organ insufficiency; (6) insufficient time or lost to follow-up. Ultimately, 79 patients were eligible for analysis in the present study (Figure 1). This study was approved by the Ethics Committee of the First Affiliated Hospital of Nanjing Medical University (approval number: No.2018-SR-218.A1). Informed consent was obtained from every participant at the time of renal biopsy.

### Clinical and Laboratory Parameters

The collection of complete clinical information of enrolled patients was performed at the time of renal biopsy, including sex, age, diabetes duration, smoking habits, blood pressure, body mass index, diabetic retinopathy, diabetic neuropathy, serum albumin, serum creatinine, hemoglobin A1c (HbA1c), estimated glomerular filtration rate (eGFR), cholesterol and triglycerides, serum C3 and C4 levels, C-reactive protein, 24-h urinary protein. The above serological examinations which were measured in fasting state and performed in the central laboratory in our hospital are routinely tested in clinical practice. Complement C4 levels were measured by rate scatter nephelometry (C4 kit, image-800 devices, Beckman Coulter, Brea, CA, USA). The cutoff point of C4 level was dichotomized at its median value. eGFR was calculated by the equation of Chronic Kidney Disease Epidemiology Collaboration (CKD-EPI) (21). The occurrence of patient visits were at intervals of 3–6 months during the follow-up. During each visit, the above clinical parameters were collected.

### Renal Histopathology

Routine examination of every renal biopsy specimen was performed by light microscopy, electron microscopy and immunofluorescence. All patients met the standard classification criteria for a pathologic classification based on histological scores for glomerular, tubulointerstitial and vascular lesions (20). Semi-quantitative scores for interstitial fibrosis and tubular atrophy (IFTA) were obtained according to the affected proportion of the tubulointerstitial compartment (0: none, 1: < 25%, 2: 25–50%, 3: > 50%), and the scale of interstitial inflammation (0: absent, 1: infiltration only in areas related to IFTA, 2: infiltration in areas without IFTA). Scores for vascular lesions were based on the existence of large-vessel arteriosclerosis and arteriolar hyalinosis (20). Semi-quantitative rank for the intensity of the complement components (including C4c, C1q and C3c) staining in each renal tissue section by direct immunofluorescence graded on a scale of 0–4 + as described previously (1). Positive complement deposition was defined as 1+ or higher grade fluorescence in all patients. Iteratively review of any scoring differences between two pathologists was performed until a consensus was reached. Quantitative analysis of the staining was measured by Image-Pro Plus 6.0 and presented as a value of integrated optical density (IOD).

### Statistical Analysis

Continuous variables were reported as either mean (standard deviation, SD) or median (interquartile range, IQR). Categorical variables were described using as counts (n) and percentages (%). Normally distributed data were analyzed by one-way analysis of variance (ANOVA) for quantitative parameters of intergroup differences, while non-normal variables were compared by Kruskal–Wallis or Mann–Whitney *U*-tests as appropriate. Differences of qualitative data were accessed using the *x*^2^ test or Fisher's exact test. Renal survival were compared with the log-rank test and evaluated by Kaplan-Meier survival curves. The independent risk factors of prognosis were assessed via multivariate Cox analysis. Pearson or Spearman correlation test was performed as the data indicated to evaluate the correlations between serum C4 and clinical parameters. To explore the predictive value of C4 protein in the progression of DKD, we used receiver-operating characteristic (ROC) curve analysis. A two-sided *p* < 0.05 was considered statistically significant. All statistics were analyzed in IBM SPSS Statistics for Windows (Version 20.0).

## Results

### DKD Patients' General Clinical Data

As shown in Table 1, of our 79 patients with DKD, male were accounted for 64 and female for 15, and aged 50.91 ± 11.34 years. Their diabetes duration was 11.0 ± 6.23 years. The mean follow-up period was 21.85 ± 16.32 months. During follow-up, 32 patients (40.5%) progressed to ESRD and 8 patients (10.1%) progressed to doubling of creatinine level. There were 4 patients (5.1%) were in class IIa, 17 (21.5%) in class IIb, 41 (51.9%) in class III, and 17 (21.5%) in class IV in terms of the glomerular classification (20).

**Table 1:** General clinical data for all enrolled DKD patients.

**Parameter**	**Values**
Patients (n)	79
Gender (male/female)	64/15
Age (years)	50.91 ± 11.34
Duration of diabetes (years)	11.0 ± 6.23
Glycosylated hemoglobin (%)	7.69 ± 1.76
Serum albumin (g/L)	31.44 ± 6.93
TG (mmol/L)	1.54 (1.07,2.3)
TC (mmol/L)	5.37 ± 1.81
LDL-C	3.47 ± 1.29
HDL-C	1.05 (0.85,1.36)
Scr (umol/L)	112.6 (90.8,175.4)
Urinary protein (g/d)	2.93 (1.84,6.96)
eGFR (ml/min/1.73 m^2^)	61 (37,82)
**Glomerular class**	
I	0 (0)
IIa	4 (5.1%)
IIb	17 (21.5%)
III	41 (51.9%)
IV	17 (21.5%)
Progression to ESRD (%)	32 (40.5%)
Progression to doubling of creatinine (%)	8 (10.1%)

*DKD, diabetic kidney disease; eGFR, estimated glomerular filtration rate; Scr, serum creatine; HDL-C, high-density lipoprotein cholesterol; LDL-C, low-density lipoprotein cholesterin; TG, triglyceride; TC, total cholesterol; ESRD, end stage renal disease*.

*Data were presented as the mean ± standard or as medians (IQR). A two-tailed p < 0.05 was considered statistically significant*.

### Comparison of Clinical Manifestations

**Figure 2:**
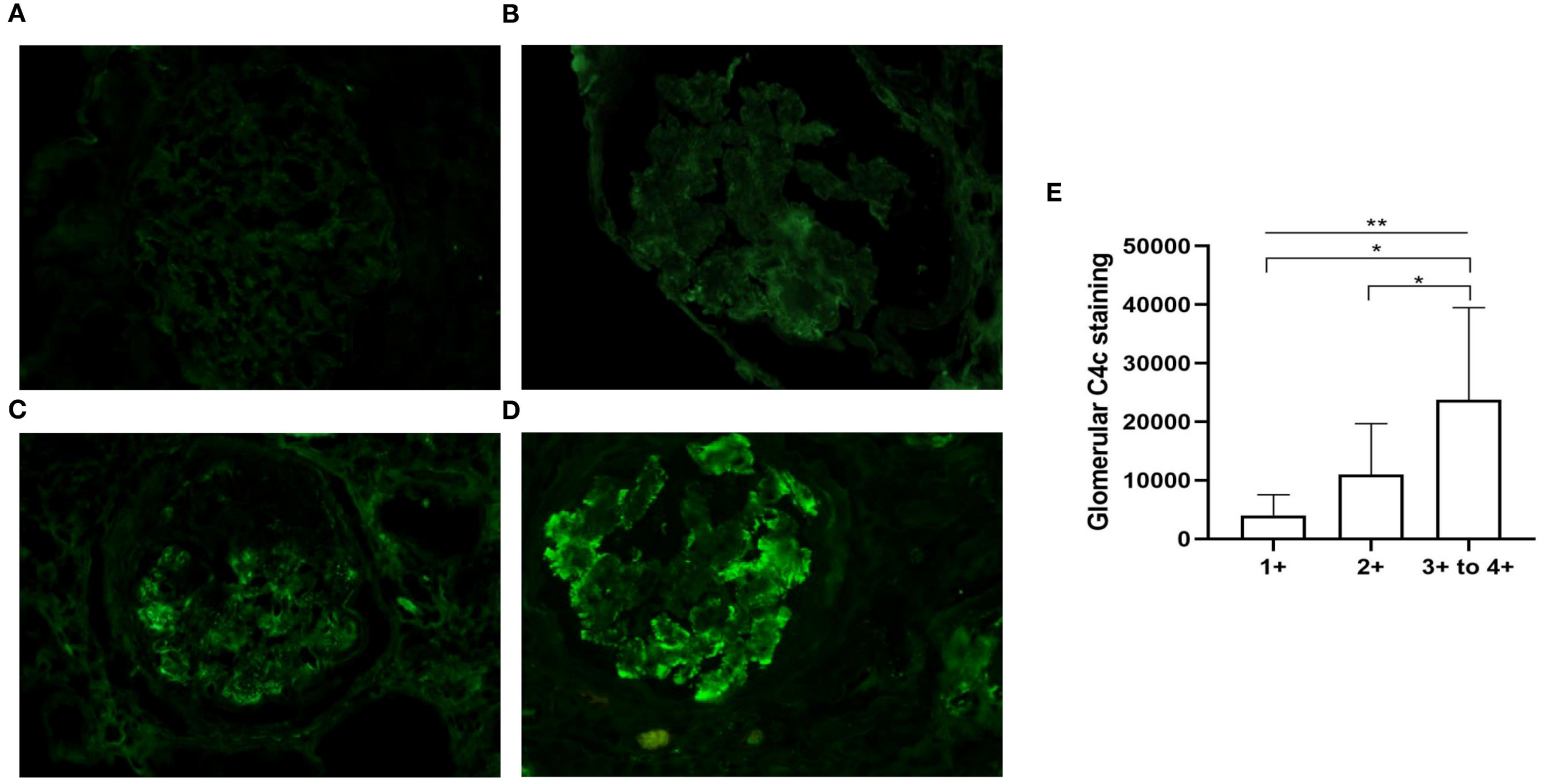
Representative immunofluorescent staining for glomerular C4c in patients with DKD. **(A)** There is no C4c staining (equal to background, C4c – to ±). **(B)** Weak and segmental staining of C4c (C4c +). **(C)** Clearly positive C4c staining along the glomerular capillary walls (C4c 2+). **(D)** Granular deposition of C4c staining along the glomerular tuft (C4c 3+ to 4+). Magnification × 400. **(E)** Quantitative analysis of glomerular C4c expression scaling 1+-4+ was performed using Image-Pro Plus 6.0 software. *p < 0.05; **p < 0.01 across the 3 groups.

On direct immunofluorescence microscopy, C4c was detected in specimens from 20/79 (25.3%) patients. C4c deposition was observed in glomerular capillary walls, Bowman's capsule and mesangium in 10/20, 1/20, and 9/20 patients, respectively. Representative fluorescence images and quantified values were presented in Figure 2. Compared with those without glomerular C4c deposition, patients with glomerular C4c deposition had significantly higher levels of urinary protein (median: 6.82 g/24 h, IQR: 2.27, 12.14 vs. 2.62 g/24 h, IQR: 1.77, 5.73; *p* = 0.008) and triglyceride (TG) (median: 1.97 mmol/L, IQR: 1.4, 3.7 vs. 1.53 mmol/L, IQR: 1.05, 1.89; *p* = 0.029), but significantly lower levels of serum albumin (28.69 ± 7.04 g/L vs. 32.38 ± 6.69 g/L; *p* = 0.039) (Table 2). Based on the glomerular complement staining patterns, patients with C4c combined C3c and C1q deposition had heavier proteinuria (median: 7.3 g/24 h, IQR: 2.68, 13.01) than those with C4c combined only C3c or C1q deposition (median: 5.59 g/24 h, IQR: 1.78, 9.56) or with negative C4c deposition (median: 2.62 g/24 h, IQR: 1.77, 5.73) (*p* = 0.021, Table 3).

**Table 2:** Baseline clinical findings in groups stratified according to the median values of serum C4 and glomerular C4c deposition on renal histology.

**Parameter**	**With C4c deposition** **(*n* = 20)**	**Without C4c deposition** **(*n* = 59)**	***p-*value**	**Lower C4 level** **(*n* = 40)**	**Higher C4 level** **(*n* = 39)**	***p-*value**
Age (years)	48.55 ± 9.64	51.71 ± 11.83	0.284	51.7 ± 11.74	50.1 ± 11.01	0.535
Gender (male)	16 (80%)	48 (81.4%)	1.000	31 (77.5%)	33 (84.6%)	0.420
T2DM (%)	20 (100%)	59 (100%)		40 (100%)	39 (100%)	
Biopsy-proven	20 (100%)	59 (100%)		40 (100%)	39 (100%)	
Duration of diabetes (years)	10.55 ± 6.56	11.16 ± 6.16	0.710	11.13 ± 6.52	10.88 ± 5.99	0.860
Diabetic retinopathy (%)	7 (35%)	19 (32.2%)	0.818	11 (27.5%)	15 (38.5%)	0.300
Diabetic neuropathy (%)	1 (5%)	4 (6.8%)	1.000	4 (10%)	1 (2.6%)	0.371
BMI (kg/m^2^)	26.21 ± 4.27	24.49 ± 3.69	0.087	25.11 ± 4.75	24.73 ± 2.81	0.663
SBP (mm Hg)	145.55 ± 16.05	144.95 ± 23.03	0.914	145.23 ± 21.04	144.97 ± 22.01	0.959
DBP (mm Hg)	87.4 ± 12.08	84.03 ± 12.24	0.289	87.38 ± 12.26	82.33 ± 11.77	0.066
Hypertension (%)	14 (70%)	43 (72.9%)	0.804	28 (70%)	29 (74.4%)	0.666
Cardiovascular diseases (%)	0 (0)	6 (10.2%)	0.481	4 (10%)	2 (5.1%)	0.695
Urinary protein (g/d)	6.82 (2.27, 12.14)	2.62 (1.77, 5.73)	**0.008**	2.71 (1.79, 5.68)	4.13 (1.87, 8.72)	0.178
eGFR (ml/min/1.73 m^2^)	47.5 (28.5, 82.75)	65 (38, 81)	0.185	65 (36.5, 82.5)	55 (37, 82)	0.655
Scr (umol/L)	137.45 (90.75, 214.15)	110.4 (90.8, 159.5)	0.127	108.6 (91.73, 167.58)	124.5 (89.2, 175.9)	0.441
Serum albumin (g/L)	28.69 ± 7.04	32.38 ± 6.69	**0.039**	31.83 ± 6.45	31.05 ± 7.44	0.624
HbA1c (%)	7 (6.2, 7.98)	7.3 (6.8, 8.1)	0.118	7.2 (6.63, 8.3)	7.2 (6.7, 8.1)	0.879
TG (mmol/L)	1.97 (1.4, 3.7)	1.53 (1.05, 1.89)	**0.029**	1.43 (1.11 1.87)	1.74 (1, 2.55)	0.283
TC (mmol/L)	5.70 ± 1.09	5.26 ± 2.0	0.214	5.43 ± 1.85	5.31 ± 1.79	0.768
LDL-C	3.74 ± 0.93	3.38 ± 1.39	0.283	3.51 ± 1.31	3.43 ± 1.28	0.788
HDL-C	1.13 (0.89, 1.39)	1.04 (0.85, 1.36)	0.701	1.06 (0.85, 1.39)	1.04 (0.87, 1.32)	0.930
Hemoglobin (g/L)	106.4 ± 14.72	110.09 ± 23.05	0.505	111.13 ± 19.51	107.13 ± 22.94	0.407
IgA (mg/dl)	232.5 (193, 259.75)	223 (185, 309)	0.644	227 (192.75, 324.75)	226 (176, 268)	0.427
IgG (mg/dl)	909.15 ± 407.69	1,007.07 ± 324.88	0.279	1,030.4 ± 381.54	932.9 ± 305.98	0.215
Complement C3 (mg/dl)	109.59 ± 15.44	109.56 ± 20.47	0.996	100.5 ± 15.42	118.87 ± 18.43	**<0.001**
Complement C4 (mg/dl)	28.97 ± 8.59	28.89 ± 8.95	0.972	21.98 ± 4.86	36.03 ± 5.68	**<0.001**
CKD stage (1/2/3a/3b/4/5)	3/4/3/5/5/0	12/21/7/14/5/0	0.092	6/16/5/8/5/0	9/9/5/11/5/0	0.809
**Pathological characteristics**						
Glomerular class (I/IIa/IIb/III/IV)	0/0/3/11/6	0/4/14/30/11	0.118	0/4/8/19/9	0/0/9/22/8	0.6
IFTA Score (0/1/2/3)	0/1/7/12	1/15/19/24	0.051	0/9/13/18	1/7/13/18	0.907
Interstitial inflammation score (0/1/2)	0/6/14	4/37/18	**0.002**	3/23/14	1/20/18	0.239
Vascular lesion Score (0/1/2)	7/8/5	23/19/17	0.971	16/14/10	14/13/12	0.595
Global sclerosis (%)	37.72 ± 19.71	30.7 ± 27.46	0.296	34.62 ± 26.44	30.28 ± 25.24	0.458
Glomerular C3c deposition (%)	15 (75%)	27 (45.8%)	**0.024**	26 (65%)	16 (41%)	**0.033**
Glomerular C1q deposition (%)	19 (95%)	19 (32.2%)	**<0.001**	21 (52.5%)	17 (43.6%)	0.428
Glomerular IgG deposition (%)	7 (35%)	17 (28.8%)	0.603	15 (37.5%)	9 (23.1%)	0.163
Glomerular IgM deposition (%)	20 (100%)	49 (83.1%)	0.114	36 (90%)	33 (84.6%)	0.703
Progression to ESRD (%)	11 (55%)	21 (35.6%)	0.127	15 (37.5%)	17 (43.6%)	0.581
Progression to doubling of creatinine (%)	1 (5%)	7 (11.9%)	0.652	4 (10%)	4 (10.3%)	1.000
**Therapy**						
RAAS inhibitor (%)	14 (70%)	44 (74.6%)	0.689	27 (67.5%)	31 (79.5%)	0.228
Oral hypoglycemic agents (%)	8 (40%)	29 (49.2%)	0.478	19 (47.5%)	18 (46.2%)	0.905
Insulin therapy (%)	19 (95%)	43 (72.9%)	0.078	30 (75%)	32 (82.1%)	0.446
Statins (%)	10 (50%)	22 (37.3%)	0.317	14 (35%)	18 (46.2%)	0.313

*ESRD, end stage renal disease; T2DM, type 2 diabetes mellitus; BMI, Body mass index; SBP, systolic blood pressure; DBP, diastolic blood pressure; eGFR, estimated glomerular filtration rate; HbA1c, Hemoglobin A1c; Scr, serum creatine; HDL-C, high-density lipoprotein cholesterol; LDL-C, low-density lipoprotein cholesterin; TG, triglyceride; TC, total cholesterol; CKD, chronic kidney disease; RAAS, Renin-Angiotensin-Aldosterone System; C1q, complement 1q; C3, complement 3; C4, complement 4; IgG, immunoglobulin G; IgM, immunoglobulin M*.

*Continuous variables were presented as means ± standard deviation or medians (interquartile range), categorical variables as n (%). The p value with statistics significant (two-tailed p < 0.05) in the tables were marked bold. Other bold values were parameters for different classification*.

**Table 3:** Characteristics of patients according to different complement deposition.

**Variables**	**C4c+C3c+C1q+** **(*n* = 15)**	**C4c+C3c+C1q-/C4+C3-C1q+** **(*n* = 5)**	**C4c-** **(*n* = 59)**	***p-*value**
**Clinical characteristics**				
Urinary protein (g/d)	7.3 (2.68, 13.01)	5.59 (1.78, 9.56)	2.62 (1.77, 5.73)^a^	**0.021**
eGFR (ml/min/1.73 m^2^)	41 (28, 82)	55 (34, 87.5)	65 (38, 81)	0.329
Scr (umol/L)	158.7 (89.2, 215.8)	122.4 (81.3, 247.85)	110.4 (90.8, 159.5)	0.233
**Pathological characteristics**				
Glomerular class (I/IIa/IIb/III/IV)	0/0/2/8/5	0/0/1/3/1	0/4/14/30/11	0.256
IFTA score (0/1/2/3)	0/1/4/10	0/0/3/2	1/15/19/24	0.122
Interstitial inflammation score (0/1/2)	0/5/10	0/1/4	4/37/18^a^	**0.006**
Vascular lesion score (0/1/2)	5/5/5	2/3/0	23/19/17	0.664
Global sclerosis (%)	39.83 ± 19.9	31.38 ± 19.81	30.70 ± 27.46	0.476

*eGFR, estimated glomerular filtration rate; Scr, serum creatine; IFTA, interstitial fibrosis and tubular atrophy; C1q, complement 1q; C3, complement 3; C4, complement 4*.

*Continuous variables were presented as means ± standard deviation or medians (interquartile range), categorical variables as n (%). The p value with statistics significant (two-tailed p < 0.05) in the tables were marked bold. Other bold values were parameters for different classification*.

a *Two-tailed p < 0.05 (compared to C4c+C3c+C1q+, post-hoc)*.

The comparison of baseline characteristics according to serum C4 groups (categorized by the median value of serum C4 levels) was also summarized in Table 2. Higher serum C4 groups tended to have higher serum C3 levels. No statistical significance was observed in other clinical parameters between the lower and higher C4 groups.

On further investigating patients with different serum C4 levels combined with positive/negative glomerular C4c deposition (groups: higher C4 level with glomerular C4c deposition, lower C4 level with glomerular C4c deposition, higher C4 level with no glomerular C4c deposition, lower C4 level with no glomerular C4c deposition), significant differences in urinary protein (*p* = 0.006) were also found in all four groups (Table 4). Further analyses revealed that, compared with patients with higher serum C4 levels and positive glomerular C4c staining, those with negative glomerular C4c deposition regardless of serum C4 concentration had significantly lower 24-h urinary protein levels (*p* < 0.01, *post-hoc*, Table 4).

**Table 4:** Characteristics of patients according to C4c deposition and/or C4 levels.

**Variables**	**Lower C4 level & no C4c deposition** **(*n* = 29)**	**Lower C4 level & C4c deposition** **(*n* = 11)**	**Higher C4 level & no C4c deposition** **(*n* = 30)**	**Higher C4 level &** **C4c deposition** **(*n* = 9)**	***p-*value**
**Clinical characteristics**					
Urinary protein (g/d)	2.74 (1.69, 4.88)^a^	2.68 (1.84, 11.51)	2.49 (1.77, 6.51)^a^	7.3 (6.04, 19.58)	**0.006**
eGFR (ml/min/1.73 m^2^)	68 (39, 80.5)	52 (30, 89)	62 (37.75, 91.25)	41 (27, 68.5)	0.495
Scr (umol/L)	103.9 (92.35, 137.55)	122.4 (81.4, 209.2)	113.45 (86.48, 175.53)	158.7 (106.85, 219)	0.38
**Pathological characteristics**					
Glomerular class (I/IIa/IIb/III/IV)	0/4/5/13/7	0/0/3/6/2	0/0/9/17/4	0/0/0/5/4	0.158
IFTA Score (0/1/2/3)	0/8/9/12	0/1/4/6	1/7/10/12	0/0/3/6	0.242
Interstitial inflammation score (0/1/2)	3/18/8^a^	0/5/6	1/19/10^b^	0/1/8	**0.006**
Vascular lesion Score (0/1/2)	14/8/7	2/6/3	9/11/10	5/2/2	0.352
Global sclerosis (%)	33.83 ± 28.32	36.69 ± 21.8	27.67 ± 26.74	38.97 ± 18.04	0.583

*eGFR, estimated glomerular filtration rate; Scr, serum creatine; IFTA, interstitial fibrosis and tubular atrophy; C4, complement 4*.

*Continuous variables were presented as means ± standard deviation or medians (interquartile range), categorical variables as n (%). The p value with statistics significant (two-tailed p < 0.05) in the tables were marked bold. Other bold values were parameters for different classification*.

a *Two-tailed p < 0.01 (compared to Higher C4 level & C4c deposition, post-hoc)*.

b *Two-tailed p < 0.05 (compared to Higher C4 level & C4c deposition, post-hoc)*.

### Comparison of Renal Histopathology

Of the 20 patients with glomerular C4c deposition, 15 (75%) also showed glomerular C3c deposition and 19 (95%) with C1q deposition, which showed significant difference compared to patients without C4c deposits (*p*=0.024, *p*<0.001; respectively). However, the two groups showed comparable proportion of glomerular IgG and IgM deposition. Moreover, comparing patients with vs. without C4c deposition, the former had significantly higher interstitial inflammation score (*p* = 0.002, Table 2). According to glomerular complement staining patterns, compared to those patients lacking glomerular C4c deposition, patients with glomerular C4c accompanied by C3c and C1q deposition showed significantly higher interstitial inflammation score (*p* < 0.05, *post-hoc*; Table 3).

Compared with the patients with higher C4, patients with lower C4 levels had a significant greater proportion of C3c deposition (65 vs. 41%; *p* = 0.033; Table 2). There were no differences in other pathological parameters between the two groups. On the other hand, on further investigations of patients in strata of composite serum C4 levels (higher/lower) and glomerular C4c deposition (positive/negative), significant differences in interstitial inflammation score (*p* = 0.006) were observed in those four groups (Table 4). More specifically, those without glomerular C4c deposits no matter the serum C4 levels had significantly lower interstitial inflammation score compared to patients with higher serum C4 levels and positive glomerular C4c staining (*p* < 0.05, *p* < 0.01, respectively; *post-hoc*; Table 4).

### Associations of C4 With Renal Outcomes of Patients With DKD

**Figure 3:**
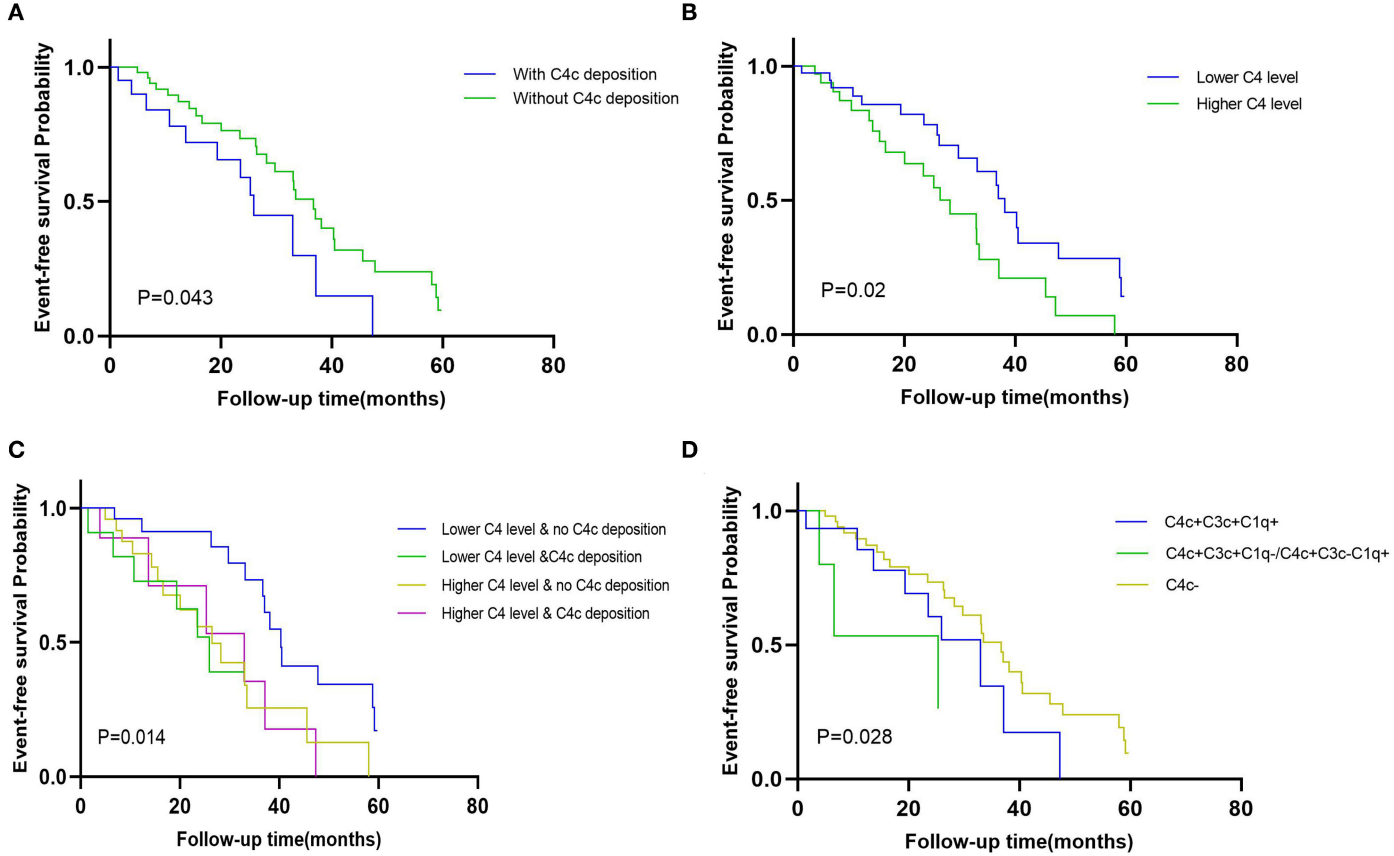
The event-free survival probability of the Kaplan-Meier analysis. Kaplan-Meier curves comparison when dividing patients in strata of **(A)** glomerular C4c deposition pattern (with C4c deposition vs. without C4c deposition) (*p* = 0.043); **(B)** median values of serum C4 (higher serum C4 level vs. lower serum C4 level) (*p* = 0.02); **(C)** composite serum and glomerular C4 levels (positive/negative glomerular C4c and higher/lower C4 levels) (*p* = 0.014); and **(D)** combined glomerular complement complex patterns (positive glomerular C4c with C3c and C1q deposition vs. positive glomerular C4c with only C3c or C1q deposition vs. negative glomerular C4c deposition) (*p* = 0.028).

In the Kaplan-Meier survival analysis (Figure 3), the event-free survival probability in patients with glomerular C4c deposition was significantly lower than those lacking C4c deposition (*p* = 0.043, Figure 3A), which suggested patients with glomerular C4c deposition had a worse renal outcome. Besides, patients with lower serum C4 levels had higher event-free survival probabilities than those with higher C4 levels (*p* = 0.02, Figure 3B). Likewise, dividing patients in strata of composite serum and glomerular C4c levels (higher/lower C4 level with glomerular C4c deposition, higher/lower C4 level with no glomerular C4c deposition), the event-free survival probability was the highest in patients with lower serum C4 levels and negative glomerular C4c deposition (*p* = 0.014, Figure 3C). Furthermore, we compared renal outcomes according to the glomerular complement deposits patterns. Patients with combined glomerular C4c, C3c, and C1q deposition (C4c + C3c + C1q) had poorer renal outcome than that of those with negative C4c deposition, yet better to that of patients with both C4c and only one other complement deposition (C4c + C3c, C4c + C1q) (*p* = 0.028, Figure 3D).

Moreover, serum levels of C4 positively associated with urinary protein (*r* = 0.327, *p* = 0.003) and serum C3 levels (*r* = 0.580, *p* < 0.001). No significant relationship was observed between serum C4 levels and glomerular DKD lesions, IFTA and Interstitial inflammation score, or vascular lesions (Table 5).

**Table 5:** Correlations between serum C4 levels and clinical characteristics.

**Parameters**	**Serum C4 levels**	
	***r***	***p***
24 h urinary protein	0.327	**0.003**
eGFR	−0.152	0.181
Scr	0.208	0.066
Serum albumin	−0.106	0.352
HbA1c	−0.035	0.757
Serum C3 levels	0.580	**<0.001**
Hemoglobin	−0.006	0.958
CRP	−0.050	0.662
Serum IgG	−0.060	0.599
Glomerular class	0.152	0.183
IFTA Score	0.101	0.374
Interstitial inflammation score	0.186	0.100
Vascular lesion score	0.054	0.635
Global sclerosis	−0.017	0.881

*eGFR, estimated glomerular filtration rate; HbA1c, Hemoglobin A1c; CRP, C-reactive protein; Scr, serum creatine; IFTA, interstitial fibrosis and tubular atrophy; C3, complement 3; C4, complement 4; IgG, immunoglobulin G*.

*The p value with statistics significant (two-tailed p < 0.05) in the tables were marked bold. Other bold values were parameters for different classification*.

Furthermore, a Cox proportional hazards model were used to determine the risks for composite endpoint (Table 6). Univariate Cox regression analysis identified combined serum and glomerular C4 as a risk factor for renal survival in DKD patients (HR 1.477, 95% CI [1.116, 1.955], *p* = 0.006). Higher concentration of serum C4 level (HR1.059, 95% CI [1.021, 1.098], *p* = 0.002) and higher intensity of glomerular C4c deposits (HR1.505, 95% CI [1.061, 2.135], *p* = 0.022) predicted unfavorable renal outcome, as did age (HR0.969, 95% CI [0.943–0.996], *p* = 0.024), diabetic neuropathy (HR 0.117, 95% CI [0.015, 0.888], *p* = 0.038), serum C3 levels (HR 1.024, 95% CI [1.007,1.043], *p* = 0.007), serum creatinine (HR 1.007, 95% CI [1.003-1.011], *p* = 0.001) and 24 h urinary protein (HR 1.161, 95% CI [1.096–1.230], *p* < 0.001) in DKD patients. As respect to pathological features, combined glomerular C4c and IgM, and interstitial inflammation were significantly associated with the development of DKD (HR 0.704, 95% CI [0.509, 0.973], *p* = 0.033; HR 1.946, 95% CI [1.102–3.435], *p* = 0.022; respectively). After adjustment for the baseline age, sex, duration of diabetes, diabetic neuropathy, eGFR, serum C4, serum C3 and interstitial inflammation, glomerular C4c remained an independent risk factor for renal survival by multivariate Cox regression (HR 1.584, 95%CI [1.001, 2.508], *p* = 0.0497) in DKD patients. However, the serum C4 level was not an independent risk factor for the renal outcomes after adjustment (HR 1.020, 95% CI [0.971, 1.072], *p* = 0.423). Moreover, baseline age (HR0.947, 95% CI [0.914, 0.982], *p* = 0.003) and diabetic neuropathy (HR 0.057, 95% CI [0.006, 0.557], *p* = 0.014) were also independent risk factors for renal dysfunction.

**Table 6:** Risk factors for renal endpoint determined by univariate/multivariate COX hazard analysis in DKD.

**Parameters**	**Univariate**		**Multivariate**	
	**HR (95% CI)**	***p*-value**	**HR (95% CI)**	***p*-value**
Age	0.969 (0.943, 0.996)	**0.024**	0.947 (0.914, 0.982)	**0.003**
Sex, male	0.873 (0.382, 1.991)	0.746	0.883 (0.371, 2.103)	0.778
Duration of diabetes	0.979 (0.931, 1.028)	0.393	1.058 (0.995, 1.126)	0.073
Diabetic retinopathy	0.834 (0.411, 1.692)	0.615	–	
Diabetic neuropathy	0.117 (0.015, 0.888)	**0.038**	0.057 (0.006, 0.557)	**0.014**
HbA1c	0.906 (0.735, 1.118)	0.358	–	
Urinary protein	1.161 (1.096, 1.230)	**<0.001**	–	
eGFR	0.992 (0.980, 1.005)	0.227	0.994 (0.982, 1.007)	0.387
Serum creatinine	1.007 (1.003, 1.011)	**0.001**	–	
Serum C4	1.059 (1.021, 1.098)	**0.002**	1.020 (0.971, 1.072)	0.423
Serum C3	1.024 (1.007, 1.043)	**0.007**	1.015 (0.991, 1.040)	0.222
Glomerular C4c intensity, per +	1.505 (1.061, 2.135)	**0.022**	1.584 (1.001, 2.508)	**0.0497**
Combined serum and glomerular C4	1.477 (1.116, 1.955)	**0.006**	–	
Combined glomerular C4c and C3c	0.809 (0.603, 1.085)	0.157	–	
Combined glomerular C4c and C1q	0.777 (0.594, 1.018)	0.067	–	
Combined glomerular C4c and IgG	0.768 (0.569, 1.035)	0.083	–	
Combined glomerular C4c and IgM	0.704 (0.509, 0.973)	**0.033**	–	
Glomerular class	1.113 (0.778, 1.593)	0.557	–	
Interstitial inflammation, per +	1.946 (1.102, 3.435)	**0.022**	1.210 (0.505, 2.9)	0.668
IFTA, per +	1.402 (0.973, 2.020)	0.069	–	
Vascular lesion	1.180 (0.828, 1.681)	0.359	–	

*eGFR, estimated glomerular filtration rate; HbA1c, Hemoglobin A1c; Scr, serum creatine; IFTA, interstitial fibrosis and tubular atrophy; C1q, complement 1q; C3, complement 3; C4, complement 4; IgG, immunoglobulin G; IgM, immunoglobulin M; CI, confidence interval; HR, hazard ratio*.

*The *p* value with statistics significant (two-tailed p < 0.05) in the tables were marked bold. Other bold values were parameters for different classification*.

**Figure 4:**
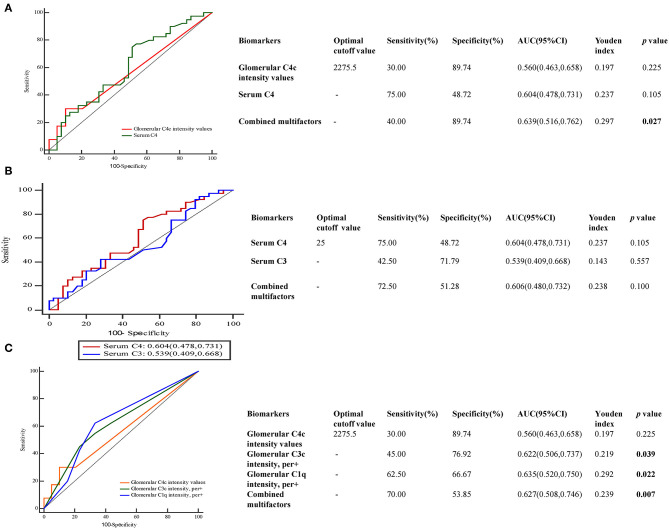
Predictive performances of variables for DKD prognosis evaluated by ROC curves. ROC curve of **(A)** glomerular C4c intensity and serum C4 levels **(B)**, serum C3 and C4 levels **(C)**, glomerular C4c, C3c, and C1q for DKD development. The AUC and the cutoff value of glomerular C4c intensity values and serum C4 levels were presented in the separate table. AUC, the area under the ROC curve; ROC, receiver operating characteristic.

Next, we performed receiver-operating characteristic (ROC) curves to determine the predictive value of C4 protein in the progression of DKD (Figure 4). The predictive values for glomerular C4c intensity values, serum C4 levels and combined multifactors were 0.560, 0.604, and 0.639, respectively (Figure 4A). As shown in Figure 4B, the optimal cutoff value of serum C4 levels for predicting DKD development was 25 mg/dl with high sensitivity (75%) but low specificity (48.72%), as calculated by obtaining the best Youden index. And the sensitivity and specificity for serum C3 levels were calculated as 42.5 and 71.79%, respectively. In addition, we compared the value of glomerular C4c in the prediction of DKD prognosis with other well-accepted proteins including C3 and C1q. The predictive values for glomerular C4c intensity values, glomerulat C3c intensity, glomerular C1q intensity, and combined multifactors were 0.560, 0.622, 0.635, and 0.627, respectively (Figure 4C). The optimal cutoff value of glomerular C4c intensity values for predicting DKD progression was 2,275.7 pixel intensity with low sensitivity (30%) but high specificity (89.74%). Moreover, glomerular C1q combined C4c and C3c intensity were significantly associated with glomerular class. However, separate deposition of C4c or C3c showed no prominent correlation (Table 7).

**Table 7:** The relationship of C4c, C3c, and C1q deposition with glomerular class.

**Parameters**	**Glomerular class**	
	***r***	***p***
Glomerular C4c intensity, per +	0.160	0.160
Glomerular C3c intensity, per +	0.221	0.051
Glomerular C1q intensity, per +	0.250	**0.027**
Glomerular C4c+C3c intensity, per +	−0.2217	**0.0496**
Glomerular C4c+C1q intensity, per +	−0.2096	0.064
Glomerular C4c+C3c+C1q intensity, per +	−0.1923	0.090

*The p value with statistics significant (two-tailed p < 0.05) in the tables were marked bold. Other bold values were parameters for different classification*.

## Discussion

In present study, we assessed the association of glomerular C4c changes with renal prognosis in patients with DKD. We found that the patients with glomerular C4c deposition had worse renal insufficiency than those without C4c deposits, along with higher 24-h urinary protein and TG, but lower serum albumin. More than 50% of the patients with glomerular C4c had co-deposition of C3c or C1q, suggesting the activation of CP in DKD. Patients with glomerular complement complex of C4c and one or two of C3/C1q deposition had more severe proteinuria and a higher rate of DKD progression than those with negative C4c deposits shown by the Kaplan–Meier analysis. Besides, serum C4 levels positively correlated with urinary protein and serum C3 levels. More importantly, the univariate Cox analysis suggested that both the serum C4 level and glomerular C4c deposition were significantly interrelated with the renal outcomes, and further analysis demonstrated glomerular C4c deposition remained an independent risk factor after the adjustment of important clinical findings and pathological features.

C4 is an essential component of the complement system and the CP and LP involved in the development of DKD could lead to C4 deposits (8, 10). The association of C4 and other immune-mediated renal diseases has been suggested in the previous studies. C4 was closely associated with lupus nephritis (22). The role of C4 has also been implicated in the outcome of IgAN (23). Although numerous studies supported a role for components of the complement system in DKD, the relationship between C4 and the progression of DKD was less discussed. One finding of the present study was that the deposition of glomerular C4c predicted an unfavorable renal outcome. Immune complex-mediated glomerular injury has been considered as a result of the kidney which is passively targeted by circulating complement components of hepatic origin. It was identified that the local complement expansion could occur in impaired kidneys and affect the progression of renal failure and survival in animal models (1, 24). The majority of patients with glomerular C4c deposits were accompanied by deposits of C1q (95%) or C3c (75%) in our study, highlighting the significance of the CP in the pathogenesis of DKD (8). It was reported that C1q combined with several autologous proteins which were relevant to DKD (25–27). Moreover, patients and animals with DKD exhibited kidney increase in C1q and C3 (28, 29). Glomerular depositions of C1q, C4d, and C5b-9 in patients with DKD were more common than those without DKD, and glomerular depositions of C4d and C5b-9 were associated with the DKD severity (11). In our study, not only proteinuria but also the rate of DKD progression were significantly higher in biopsy-proven DKD patients with the glomerular immune complex of combined C4c with one or two of C3/C1q deposition than those with negative C4c deposits by the Kaplan-Meier survival analysis. These data reinforced the critical role of complement system in the development of DKD.

IgM is known to be able to activate classical complement pathway. Combined glomerular C4c and IgM is one of the risk factors for composite endpoints in DKD (*p* = 0.033) as demonstrated via univariate analysis in the study. Consistently, previous data demonstrated the segmental staining of mesangial and capillary IgM and C4c in the glomeruli of normal mice and human renal biopsies (30, 31). A growing body of experimental and clinical evidence suggested that IgM originated from B cells might involve in diabetic kidney injury. By generating auto-antibodies and regulating inflammatory cytokines and T cell function, human B cells were reported to be correlated with the inflammation in obesity as well as T2DM (32). The amount of intrarenal B cells was remarkably elevated and associated with urinary protein in patients with T2DM (33). Hence, therapy targeting B cells has come into focus with the evidence that anti-CD20 specific antibody improved glomerular IgM deposition and proteinuria in an adriamycin model, as well as reversed and suppressed autoimmune diabetes. Thus, we deduce that glomerular C4c with IgM deposition might help the clinicians to draw attention to cases with progression to DKD to whom B cell-targeted regimen might be effective. More detailed research is needed for better understanding of the underlying mechanism of C4 and IgM in DKD.

Higher interstitial inflammation score was a risk factor for renal survival in DKD patients in our study. Accumulating data from experimental and clinical studies, has demonstrated that inflammation in diabetic kidneys is associated with the progression of kidney diseases (34). Consistently, we observed that patients with glomerular C4c deposition predicted an unfavorable renal outcome had significantly higher interstitial inflammation score. Our results suggested that the activation of complement significantly might contribute to inflammation-mediated tissue damage. The previous study indicated that the complement system was composed of a large number of plasma proteins, mostly proteases that react with each other by proteolytic cleavage to induce inflammatory responses (35, 36). Further research is needed to elucidate the role of C4c and its signaling in the development of inflammation and fibrosis in DKD. Improved understanding of the integration and regulation of the component of the complement system in renal inflammation might provide novel, more specific therapeutic targets for the treatment of DKD.

In the present study, serum C4 levels showed positive associations with proteinuria and the rate of the progression to ESRD or doubling serum creatinine was significantly higher in T2DKD patients with higher serum C4 levels. This was consistent with the previous data that the serum C4 was significantly associated with serum C3 level and the DKD progression (12). However, another study indicated that decreased C4 levels might be a consequence of diabetes, instead of a causal factor of the disease. Discrepancies of the assessment of C4 allotypes between DM patients with and without microvascular complications have been reported in a previous study (8). That could at least partially explain the current result that serum C4 level was not emerged as an independent risk factor in DKD patients for renal prognosis after adjustment. Accordingly, the predictive strength of serum C4 levels in DKD prognosis was limited that AUC for serum C4 was calculated as 60.4% with cutoff value of 25 mg/dl. Compared with serum C3 levels, it was rather sensitive than specific. The pathogenic correlation between increased C4 level and the development of kidney lesions in DKD patients needs to be clarified in future studies.

The predictive value of combined glomerular C4c deposition and circulating C4 levels was 0.639 with low sensitivity (40%) but high specificity (89.74%). Glomerular C1q was found to be beneficial in the prediction of DKD development which achieved the predictive efficiency with AUC of 0.635, and it has been showed significantly correlation with glomerular class, compared to glomerular C4c and C3c deposition. When combined multifactors of complement deposits, the predictive values for both glomerular C4c and C3c have been boosted. Furthermore, combined C4c and C3c were significantly associated with glomerular class. However, separate deposition has showed no prominent correlation. Combined with the results that the positive rate of C4c was relative low, glomerular C4c might serve as a supplement of other components of complement systems to predict the prognosis in DKD.

There are several limitations to the present study. First, this was an observational study that could not make a causal inference. Second, this study was performed in a single center with limited sample size. Also, these T2DM patients receiving the renal biopsy might have atypical symptoms of DKD. There might be selection bias. Third, the other complements, specifically the markers of AP, C4d were not examined in present study. Finally, the tubular and vessel complement deposits were not assessed which might be also involved in the pathogenesis of DKD.

## Conclusion

In summary, we demonstrated that the glomerular C4c deposition was associated with deteriorated renal function and outcomes in patients with T2DKD. Glomerular C4c deposition was an independent risk factor that might serve as a promising and novel biomarker to predict the development of DKD.

## Data Availability Statement

The raw data supporting the conclusions of this article will be made available by the authors, without undue reservation, to any qualified researcher.

## Ethics Statement

The studies involving human participants were reviewed and approved by the Ethics Committee of the First Affiliated Hospital of Nanjing Medical University. The patients/participants provided their written informed consent to participate in this study.

## Author Contributions

SD designed and conducted the research and analyzed the data. GN and LS contributed substantially to the writing and critical review of the manuscript. JC, CZ, HZ, ZH, JQ, and XZ reviewed the manuscript. CX and YY coordinated and conceived the study as well as revised the manuscript. BZ was the guarantor of this work and had complete access to all the data in the study. All authors have read the final paper and approved the submission.

## Conflict of Interest

The authors declare that the research was conducted in the absence of any commercial or financial relationships that could be construed as a potential conflict of interest.
